# Health Tract, No. 255

**Published:** 1866-10

**Authors:** 


HEALTH TRACT, No. 255.

### EARLY RISING.

Health and long life are almost universally associated with early rising; and we are pointed to countless old people, as evidence of its good effect on the general system. Can any one of our readers on the spur of the moment, give a good conclusive reason why health should be attributed to this habit? We know that old people get up early, but it is simply because they can't sleep. Moderate old age does not require much sleep; hence, in the aged, early rising is a necessity, or convenience, and is not a cause of health in itself.—There is a larger class of early risers, very early risers, who may be truly said not to have a day's health in a year—the thirsty folks, for example, who drink liquor until midnight and rise early to get more! One of our earliest recollection is that of "old soakers" making their "devious way" to the grog-shop or tavern bar-room, before sunrise, for their morning grog. Early rising, to be beneficial, must have two concomitants; to retire early and on rising to be properly employed.—One of the most eminent divines in this country rose by daylight for many years, and at the end of that time became an invalid, has traveled the world over for health, and has never regained it, nor ever will. It is rather an early retiring that does the good, by keeping people out of those mischievous practices which darkness favors, and which need not here be more particularly referred to.

Another important advantage of retiring early is, that the intense stillness of midnight and the early morning hours favor that unbroken repose which is the all-powerful renovator of the tired system. Without, then, the accompaniment of retiring early, "early rising" is worse than useless, and is positively mischievous. Every person should be allowed to "have his sleep out;" otherwise, the duties of the day cannot properly be performed, and will be necessarily slighted, even by the most conscientious.

To all young persons, to students, to the sedentary, and to invalids, the fullest sleep that the system will take, without artificial means, is the balm of life—without it there can be no restoration to health and activity again. Never wake up the sick or infirm, or young children, of a morning—it is a barbarity; let them wake of themselves, let the care rather be to establish an hour for retiring, so early that their fullest sleep may be out before sunrise.

Another item of very great importance is, do not hurry up the young and the weakly. It is no advantage to pull them out of bed as soon as their eyes are open, nor is it best for the studious or even for the well who have passed an unusually fatiguing day, to jump out of bed the moment they wake up: let them remain without going to sleep again until the sense of weariness passes from their limbs. Nature abhors two things: violence and vacum. The sun does not break out at once into the glare of the meridian. The diurnal flowers unfold themselves by slow degree; nor fleetest beast, nor sprightliest bird, leaps at once from his resting place. By all which we mean to say, that as no physiological truth is more demonstrable, than that as the brain, and with it the whole nervous system, is recuperated by sleep, it is of the first importance, as to the well-being of the human system, that it have its fullest measure of it; and to that end, the habit of retiring to bed early should be made imperative on all children, and no ordinary event should be allowed to interfere with it. Its moral healthfulness is not less important than its physical. Many a young man, many a young woman, has taken the first step towards degradation, and crime and disease, after ten o'clock at night; at which hour, the year round, the old, the middle aged, and the young, should be in bed; and the early rising will take care of itself, with the incalculable accompaniment of a fully rested body and a renovated brain. We repeat it there is neither wisdom nor safety, nor health, in early rising in itself; but there is all of them in the persistent practice of retiring to bed at an early hour, winter and summer.

